# Cases of Intermittent Odontalgia or Neuralgia

**Published:** 1853-07

**Authors:** H. R. Smith

**Affiliations:** Terre Haute, Ind.


*Cases of Intermittent Odontalgia or Neuralgia.* By DR. H. R. SMITH, Terre Haute, Ind.

Within the past five months, I have had some very interesting cases of intermittent odontalgia, or neuralgia. And as it is a disease which frequently occurs in our western practice, (or mine at least,) I think it well worthy of some notice, from our profession.

I will relate a case in my practice, which will, in the main features, illustrate the type of the whole. For a disease so common, and one so severe, little, comparatively, has been written upon it. I have no doubt, that many teeth, that otherwise might be saved, are sacrificed, on acccount of ignorance of the cause of the pain.

Mr. G.——aged forty, a brick mason by trade, sent for me in great haste, to extract a right superior bicuspid tooth. I found him suffering under a severe paroxism of pain in the tooth and face, with a blister extending from the tooth to the ear, and, "Davis pain killer on cotton around the aching tooth."

He had been under the care of his physician for four days, who was treating him for a "cold," giving epsom salts to "cleanse the system," and morphine and gum camphor to allay the spasmodic action.

The paroxisms occurred at 8 o'clock A. M. and at 7 o'clock P. M., and lasted about two hours. During the intervals he had considerable fever. At the time I was called, the pain in the tooth had become so severe, that the physician as a last resort, advised the extraction of the tooth.

And with turnkey in hand, was about to displace the aching member, when the patient cries out, "hold on Doctor, you can't pull my tooth with that instrument, for I have had my jaw broken with it, and shall not let it go into my mouth again."

He accordingly sent for me, and on examination, I found the tooth somewhat decayed, but not into the nerve, and no marks of severe external inflammation.

But, the paroxisms returning at regular periods, satisfied me of its intermittent type. I accordingly advised 10 grains of calomel at night, and the next morning at four o'clock, 2 grains of quinine, repeating each hour, until the time for the return of the paroxism, and the night following, a mild cathartic. I saw the patient the next day, and there had been no return of the tooth-ache, nor has there as yet, it now being near six weeks.

I mention the above case, not to show any treatment for "fever and ague" but to show the importance of studying the sympathetic relations which general diseases bear to the teeth and face, and how, by finding out the cause of the tooth-ache, we may avoid extracting, aud make many teeth, which would otherwise be extracted, useful organs for life.

